# Regulation of Pattern-Recognition Receptor Signaling by HBX During Hepatitis B Virus Infection

**DOI:** 10.3389/fimmu.2022.829923

**Published:** 2022-02-17

**Authors:** Hongjuan You, Suping Qin, Fulong Zhang, Wei Hu, Xiaocui Li, Dongsheng Liu, Fanyun Kong, Xiucheng Pan, Kuiyang Zheng, Renxian Tang

**Affiliations:** ^1^ Jiangsu Key Laboratory of Immunity and Metabolism, Department of Pathogenic Biology and Immunology, Xuzhou Medical University, Xuzhou, China; ^2^ Imaging Department, The Second Affiliated Hospital of Shandong First Medical University, Taian, China; ^3^ Nanjing Drum Tower Hospital Group Suqian Hospital, The Affiliate Suqian Hospital of Xuzhou Medical University, Suqian, China; ^4^ Department of Infectious Diseases, The Affiliated Hospital of Xuzhou Medical University, Xuzhou, China; ^5^ National Demonstration Center for Experimental Basic Medical Sciences Education, Xuzhou Medical University, Xuzhou, China

**Keywords:** hepatitis B virus, retinoic acid inducible gene I (RIG-I)-like receptors, HBx, pattern recognition receptors, toll-like receptors, nod-like receptors

## Abstract

As a small DNA virus, hepatitis B virus (HBV) plays a pivotal role in the development of various liver diseases, including hepatitis, cirrhosis, and liver cancer. Among the molecules encoded by this virus, the HBV X protein (HBX) is a viral transactivator that plays a vital role in HBV replication and virus-associated diseases. Accumulating evidence so far indicates that pattern recognition receptors (PRRs) are at the front-line of the host defense responses to restrict the virus by inducing the expression of interferons and various inflammatory factors. However, depending on HBX, the virus can control PRR signaling by modulating the expression and activity of essential molecules involved in the toll-like receptor (TLR), retinoic acid inducible gene I (RIG-I)-like receptor (RLR), and NOD-like receptor (NLR) signaling pathways, to not only facilitate HBV replication, but also promote the development of viral diseases. In this review, we provide an overview of the mechanisms that are linked to the regulation of PRR signaling mediated by HBX to inhibit innate immunity, regulation of viral propagation, virus-induced inflammation, and hepatocarcinogenesis. Given the importance of PRRs in the control of HBV replication, we propose that a comprehensive understanding of the modulation of cellular factors involved in PRR signaling induced by the viral protein may open new avenues for the treatment of HBV infection.

## Introduction

Although the hepatitis B virus (HBV), a small DNA virus, has been known for more than 50 years, chronic infection caused by this virus remains a global cause of hepatitis, cirrhosis, and liver cancer, especially hepatocellular carcinoma (HCC) (1–3). It has been widely demonstrated that after recognition by pattern recognition receptors (PRRs), the virus can be restrained by the innate immune system (4–7). To date, four types of PRRs, including toll-like receptors (TLRs), cytosolic DNA sensors, retinoic acid-inducible gene I (RIG-I)-like receptors (RLRs), and NOD-like receptors (NLRs), have been discovered (8–10). Generally, once sensitized by the virus, these PRRs can initiate the activation of multiple intracellular signaling pathways to produce interferons (IFNs), which further stimulate the sensitization of the Janus kinase-signal transducer and activator of transcription (JAK-STAT) signaling to participate in the inherent antiviral immune response. Additionally, these PRRs can stimulate the production of inflammatory cytokines induced by the nuclear factor κB (NF-κB) pathway to resist the virus (11, 12). However, accumulating evidence suggests that to maintain persistent infection, the virus has evolved a variety of strategies to overcome the host antiviral responses mediated by PRR components, such as TLRs, cytosolic DNA sensors, RLRs, and NLRs, as well as their downstream pathways, including the JAK-STAT and NF-κB pathways, to facilitate viral replication and liver pathogenesis (13–16). Moreover, the exact mechanisms by which this virus evades the immune response mediated by PRRs to facilitate its persistent infection and development of different diseases remain elusive.

HBV genome contains four overlapping open reading frames (ORFs), namely S, P, C, and X. S ORF contributes to the production of large, middle, and small envelope proteins that are composed of HBsAg, preS1, or preS2 antigens; P and C ORFs encode the HBV polymerase protein, core protein, and HBeAg. Additionally, X ORF is responsible for the expression of HBX, a highly conserved nonstructural protein with 154-amino acids (17–19). Based on the studies on cell and mouse models, HBX is considered to be important for initiating and maintaining HBV replication (20, 21). Mechanistically, the viral protein in the cell cytoplasm can stimulate signal transduction pathways to facilitate HBV replication. In the nucleus, HBX can bind to the HBV covalently closed circular DNA (cccDNA) and activate the transcription of viral promoters with the help of different transcription factors and epigenetic regulatory molecules (22–24). At different stages of HBV infection, the viral protein performs various biological functions (22), participate in the growth, migration, autophagy, apoptosis, and epigenetic regulation of virus-infected hepatocytes, and play prominent roles in the development of different liver diseases, especially HCC (25–28). More importantly, there is growing evidence that HBX can regulate the expression and activity of numerous molecules in different PRR subfamilies, including TLRs, RLRs, and NLRs (29–32). In the present review, we summarize the current research on the modulation of PRR signaling induced by HBX to regulate the innate immune responses that aid in HBV replication and various biological functions that facilitate the development of HCC.

## Effect of HBX on TLR-mediated Signaling Pathways

TLRs are a large group of conserved type I transmembrane molecules. So far, 10 humans TLRs have been discovered that identify specific pathogen-associated molecular patterns (PAMPs) (33, 34). Among the identified TLRs, TLR1-2 and TLR4-6 are expressed on the cell surface, whereas TLR3 and TLR7-9 are situated on the endosomal surface. Upon activation, the TLRs (except TLR3) can activate the adaptor protein, myeloid differentiation primary response 88 (MyD88), to enhance the sensitization of the tumor necrosis factor (TNF) receptor-associated factor (TRAF) 6, which further promotes phosphorylation of the transforming growth factor-activated kinase-1 (TAK1). Next, TAK1 activates NF-κB signaling and then allows NF-κB to translocate into the nucleus to initiate the gene expression of inflammatory factors. Additionally, TAK1 activation also prompts the sensitization of mitogen-activated protein kinases (MAPK), which can cause the recruitment of the transcription factor activator protein 1 (AP-1) to upregulate the induction of inflammatory cytokines. TLR3 is unique and uses MyD88-independent signaling pathways to initiate the immune response. After TLR3 activation is triggered by double-stranded RNA (dsRNA), it can sensitize toll-interleukin-1 receptor (TIR)-domain-containing adaptor-inducing IFN-β (TRIF) to activate the protein complex composed of TRAF3 and TANK-binding kinase 1 (TBK1). These molecules activate IFN regulatory factor (IRF)-3/7 and NF-κB. TLR3 also recruits TRAF6 to phosphorylate TAK1. TAK1 sensitizes AP-1 *via* the activation of the MAPK pathway and activate NF-κB *via* the IKK-IκB complex to modulate the inflammatory reaction (33–38).

Accumulating data shows that initiating the host TLR response is a new therapeutic strategy for HBV infection (36, 37). Several studies have demonstrated that the stimulation of TLR2-5, TLR7, and TLR9 with their specific ligands can inhibit HBV replication in the cell and animal models (38). Furthermore, the potential of TLR agonists, including GS-9620, RO7020531, JNJ-64794964 (TLR7 agonists), GS-9688 (TLR8 agonist), and AIC649 (TLR9 agonist), has been investigated in clinical trials at different stages (38, 39). HBV is considered as a “stealth virus,” and the suppression of TLR signaling molecules has been reported in different kinds of cells in patients with HBV infection (40, 41). For example, decreased expression levels of TLR2 and TLR9 have been reported in the peripheral monocytes of patients with chronic hepatitis B (CHB) (42, 43). Suppressed TLR signaling molecules, such as TLR3 (44), TLR7, TLR9 (45), TRAF3, IRAK4, and IRF7 (46), were observed in the peripheral blood mononuclear cells (PBMCs) of patients with chronic HBV infection. Additionally, downregulation of TLR7 and TLR9 was also observed in the plasmacytoid dendritic cells (pDCs) in HBV-infected patients (47–49).

Furthermore, several studies have shown that multiple virus-encoded proteins, including HBsAg, HBeAg, and HBV polymerase, contribute to HBV persistence by inhibiting TLR-mediated antiviral responses (38). For instance, HBsAg can inhibit TLR3-mediated immune response in murine Kupffer cells and sinusoidal endothelial cells (50). HBeAg has been shown to restrict TLR2 expression and interact with the TIR proteins, TRAM and Mal, to suppress TLR-mediated immune responses in hepatocytes (51, 52). Meanwhile, HBV polymerase suppresses TLR3-mediated induction of IFN-β in hepatocytes by interfering with IRF3 activation (53) and inhibits MyD88 expression by blocking the nuclear translocation of STAT1 (54). Additionally, HBsAg and HBeAg can control the major vault protein (MVP)-induced IFN production by suppressing the interaction between MVP and MyD88 in liver cells (55).

In addition to viral replication, molecules involved in TLR signaling also contribute to the pathological changes in the liver caused by the virus. For example, the virus can activate B cells *via* the TLR2 signaling pathway, which may be associated with the activation of antiviral responses mediated by B cells in patients with CHB (56). Among the proteins encoded by the virus, HBeAg sensitizes macrophages *via* the TLR2 signaling pathway to exacerbate hepatic fibrosis (57). Depending on the TLR2 signaling pathway, the HBV core protein promotes the production of the inflammatory cytokines, IL-6 and TNF-α, from M2 macrophages (58). After HBeAg stimulation, upregulation of TLR4 was also observed in the monocytes of patients with CHB. Moreover, overexpression of TLR4 on monocytes mediated by HBeAg may regulate the activity of regulatory T cells, which is related to the immunotolerance of the virus infection (59). Additionally, HBsAg can enhance the invasion of HBV-associated HCC cells by upregulating TLR2 (60). HBsAg also inhibits the production of IFN-α by pDCs by decreasing TLR9 expression in pDCs, and this effect may be related to the reduced capacity antiviral immune response of pDCs in patients with CHB (61).

**Figure 1:**
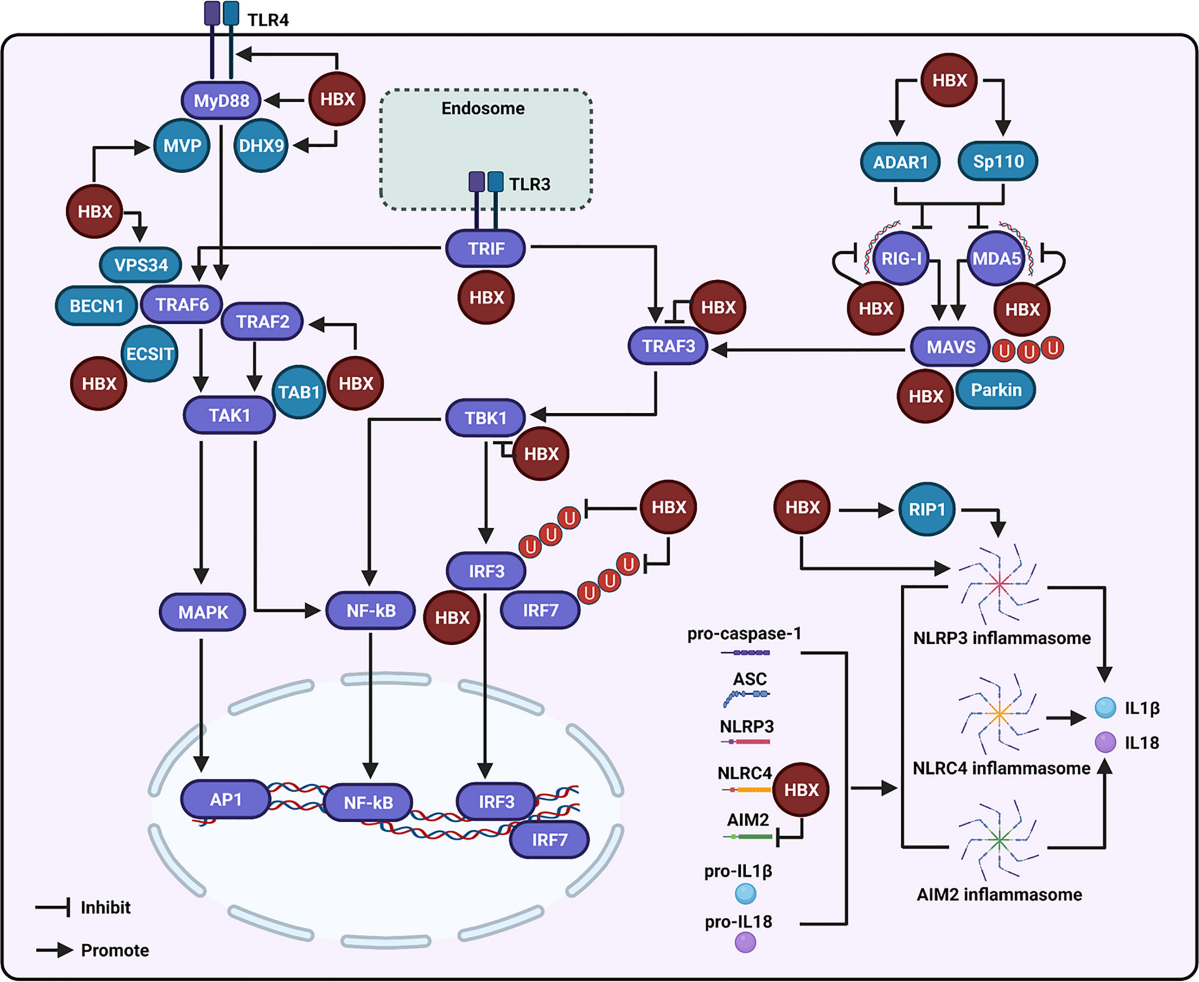
Regulation of the hepatitis B virus X protein (HBX) on the toll-like receptor (TLR), retinoic acid inducible gene I (RIG-I)-like receptor (RLR), and NOD-like receptor (NLR) signaling-associated molecules. During the modulation of TLR signaling mediated by HBX, the viral protein can promote the expression of TLR4 and myeloid differentiation primary response 88 (MyD88), and enhance the levels of the major vault protein (MVP) and DExH-box RNA helicase 9 (DHX9), which may further interact with MyD88 to regulate the innate immune response. HBX can activate the tumor necrosis factor (TNF) receptor-associated factor (TRAF)-2/transforming growth factor-activated kinase-1 (TAK1) signaling pathway. HBX is also able to interact with TAB1, and the interaction may affect the function of TAK1. The viral protein is capable of interacting with the evolutionarily conserved signaling intermediate in Toll pathway (ECSIT), which is a partner of TRAF6. The viral protein also contributes to the interaction of vacuolar protein sorting 34 (VPS34) with Beclin 1 (BECN1) and TRAF6. HBX interacts with toll-interleukin-1 receptor (TIR)-domain-containing adaptor-inducing IFN-β (TRIF), a downstream molecule of TLR3. HBX can bind to RIG-I, melanoma differentiation-associated 5 (MDA5), and mitochondrial antiviral signaling (MAVS) to inhibit RLR signaling. Adenosine deaminase acting on RNA 1 (ADAR1) and speckled at 110 kDa (Sp110) may participate in the inhibition of RIG-I and MDA5 mediated by HBX. Parkin is involved in the degradation of MAVS induced by HBX. Besides these, HBX also interacts with TRAF3 and TBK1 to inhibit their activities. HBX can bind to IRF3 and inhibit the ubiquitination of IRF3 and IRF7 at the lysine 63 sites to suppress their activation. During the modulation of NLR signaling, HBX can activate the NLR family pyrin domain-containing 3 (NLRP-3) inflammasome. RIP1 may be involved in the activation of NLRP3 inflammasome mediated by HBX. Moreover, HBX can interact with the NLR with CARD domain-containing 4 (NLRC4) and through this interaction, HBX may influence the NLRC4 inflammasome. The viral protein can also inhibit the expression of absent-in-melanoma-2 (AIM2) and may further affect the AIM2 inflammasome.

HBX has a critical role in suppressing host innate immune response by disrupting TLR signaling to regulate viral replication. Moreover, the molecules regulated by HBX in TLR signaling contribute to virus-mediated inflammation and hepatocarcinogenesis. Among the identified TLRs, HBX was found to upregulate TLR4 in immortalized proximal tubule epithelial cells (**Figure 1**), which may be associated with the dysregulated expression of cytokines, including IL-6, TNF-α, IFN-γ, and IL-4, mediated by the viral protein (62). TLR4 is upregulated in HBV-related hepatoma cells, and promotes their growth, while inhibiting the apoptosis of these cells, by activating the extracellular signal-regulated kinase (ERK)-1/2 signaling pathway (29). HBX can interact with TLR4 in HBV-related hepatoma cells, and the physical interaction of HBX with TLR4 may contribute to the activation of ERK1/2 in hepatoma cells. To date, the effect of HBX-mediated TLR4 on HBV replication has not been well investigated. However, in response to TLR4 stimulation, HBX facilitates the migration of liver cancer cells by enhancing the interaction of vacuolar protein sorting 34 (VPS34) with the TRAF6-Beclin 1 (BECN1) complex (63), thereby increasing BECN1 ubiquitination and autophagy, a physiological process that contributes to HBV replication (64).

MyD88 can accelerate the degradation of HBV pre-genomic RNA to restrain its replication (65, 66). HBX can promote the expression of MyD88 at the transcriptional level in both liver and hepatoma cells. To date, the effect of MyD88 on HBX-mediated HBV replication has not been assessed. However, the role and associated molecular mechanisms of viral proteins in the regulation of MyD88 and its related proteins in TLR signaling to facilitate HCC development have been investigated. In particular, HBX activates the downstream signaling molecules of MyD88, including IRAK1, ERK/p38, and NF-κB, to induce the production of IL-6, a major inflammatory cytokine that facilitates the development of HCC (67). However, Wu et al. suggested that HBX was able to inhibit MyD88 promoter activity in hepatocytes during IFN-α stimulation (54). The reasons for the inconsistent results regarding the effect of HBX on the expression of MyD88 with or without exogenous stimulation should be further assessed in future studies.

MVP interacts with MyD88 to stimulate IFN production (3, 55). Liu et al. observed high levels of MVP in the liver tissues of HBV-infected patients. Researchers have also elucidated that HBX can stimulate MVP promoter activity to enhance its expression in hepatocytes. Functionally, Yu et al. found that MVP is involved in the proliferation, migration, and invasion of cells mediated by HBX, by sequestering IRF2 and enhancing the HDM2-dependent loss of P53 (68). In addition to MVP, DExH-box RNA helicase 9 (DHX9) directly binds to MyD88 to facilitate IFN production (69). A recent study showed that HBX can enhance the expression of DHX9 by inhibiting its degradation, which is regulated by MDM2. Moreover, the interaction between DHX9 and Nup98 contributes to HBX-mediated HBV replication (70). In addition, HBX is able to interact with DHX9 to downregulate the circular RNA circSFMBT2 and then release miR-665 to suppress TIMP3 expression and enhance HCC metastasis (71). However, the importance of DHX9 in modulating the HBX-mediated innate immune response has not been well investigated.

In TLR signaling, stimulation of MyD88 can induce the activation of TAK1 to initiate the inflammatory reaction and IFN production (33–35). Zhou et al. showed that HBX is capable of inducing the activation of TAK1 through TRAF2 to stimulate NF-κB and cause the upregulation of IP-10, and TRAF2 may participate in the regulation of TAK1 mediated by MyD88. Furthermore, HBX-mediated increase in IP-10 is associated with the migration of leukocytes, which may cause pathological immune injury of the liver during HBV infection (72). Activation of TAK1 is dependent on its interaction with its binding partners, TAK1-binding proteins (TAB1, TAB2, and TAB3). Based on immunoprecipitation and mass spectrometric analyses, HBX interacts with TAB1 (73). However, based on this interaction, whether HBX could regulate the function of TAK1 and then modulate the innate immune response has not been assessed so far.

Activation of TRAF6 requires oligomerization, and HBX significantly enhances TRAF6 activation by promoting its oligomerization. Furthermore, TRAF6 oligomerization facilitates its interaction with histone deacetylase 3 (HDAC3) and then promotes gene expression and protein stability of c-Myc to promote hepatocarcinogenesis (74). The evolutionarily conserved signaling intermediate in Toll pathway (ECSIT) is a partner of TRAF6 and activates both NF-κB and AP-1. Chen et al. revealed that HBX could interact with ECSIT to increase NF-κB activation, leading to the induction of IL-10, an inflammation-related cytokine (75). In addition, TRIF is a vital adaptor protein that initiates TLR3-mediated innate immune signaling. Hong et al. showed that HBX can enhance TRIF protein degradation to evade the innate immune response and facilitate HBV replication (76). Taken together, these studies suggest that HBX can regulate the expression of multiple molecules in TLR signaling to inhibit the immune response and facilitate viral replication. Moreover, the viral protein is also capable of utilizing molecules in the TLR signaling pathway to regulate inflammation and enhance the development of HCC.

## Role of HBX in RLR-mediated Signaling Pathways

The DExD/H box RNA helicases, RIG-I and melanoma differentiation-associated gene 5 (MDA5), participate in the activation of RLR signaling (77, 78). These two molecules recognize dsRNAs from various viruses in the cytoplasm. Once RIG-I and MDA5 are sensitized by dsRNA, they can activate mitochondrial antiviral signaling (MAVS), which occurs on the mitochondrial membrane. MAVS further initiates TRAF3 activation. TRAF3 sensitizes TBK1 to activate IRF3/7 and leads to the production of IFN (8, 79).

Current evidence shows that RIG-I can recognize HBV pregenomic RNA and suppress HBV cccDNA, thereby activating the innate immune response to inhibit its replication (80–82). Similar to TLR, drugs activating RLR signaling also underwent a phase II trial to investigate the therapeutic effects of PLR agonists on HBV inhibition in clinical settings (13). It was found that the virus could escape immune reactions by regulating RLR signaling (13). For example, no significant increase in RIG-I and MDA5 levels was found in the liver tissues of patients with CHB (83). To benefit from persistent HBV infection, the virus uses N^6^-methyladenosine modification to block the recognition of viral RNA mediated by RIG-I signaling (84). The virus also restricts immune signaling mediated by RIG-I by inducing miR146a (85). Besides these, based on its polymerase protein, HBV can inhibit RIG-I-mediated IFN induction by inhibiting the interaction between TBK1 and DDX3 (53).

HBX plays a central role in the regulation of viral replication by dysregulating RLR signaling. For example, to facilitate HBV replication, HBX can suppress RIG-I, MDA5, and MAVS-mediated activation of IFN-β promoters (31, 86) (**Figure 1**). Furthermore, residues Asn118 and Glu119 of HBX were found to be critical for the viral protein-mediated suppression of RIG-I-MAVS signaling (87). Mechanistically, HBX binds to RIG-I and MDA5 (31, 86). Similarly, HBX also interacts with MAVS and disrupts the interactions between MAVS and other proteins within the MAVS-associated complex, including RIG-I, MDA5, and TBK1, to suppress IFN-β promoter sensitization (31). Furthermore, the interaction of HBX with MAVS promoted protein degradation to block IFN-β production (88). In addition, HBX-induced Parkin is capable of binding to the accumulating unanchored linear polyubiquitin chains on MAVS *via* the linear ubiquitin assembly complex (LUBAC) to disrupt MAVS signalosome and abate IRF3 sensitization (89).

Studies shown that adenosine deaminases acting on RNA 1 (ADAR1), RNA-editing enzymes that convert adenosine to inosine in duplex RNA regions, are vital cellular factors controlling the innate immune response mediated by endogenous RNAs. Relying on the transcription factor YY1, HBX was observed to accelerate ADAR1 expression in a dose-dependent manner to block the transcriptional levels of MDA5 and RIG-I, and inhibit the recognition of HBV RNA mediated by these two molecules in hepatocytes (90). The transcription factor, speckled at 110 kDa (Sp110), can control the levels of RIG-I and MDA5. HBX interacts with Sp110 and may modulate the production of RIG-I and MDA5 to control the innate immune response (91). In addition to RIG-I, MDA5, and MAVS, HBX interacts with TRAF3, TRIF, TBK1, and IRF3 to inhibit IFN induction (86). Furthermore, HBX acts as a deubiquitinating enzyme to suppress IRF3 and IRF7 ubiquitination with lysine 63-linked chains and attenuates their activities (86). The expression levels of TBK1 are increased in the liver cancer tissue samples. In particular, HBX upregulates the expression of TBK1 to enhance NF-κB activation (92). However, the biological role of HBX-mediated TBK1 has not yet been investigated.

## Function of HBX in NLR-mediated Signaling Pathways

The NLR family contains a variety of cytoplasmic sensors, such as the NLR family pyrin domain-containing (NLRP)-1, NLR with CARD domain-containing 4 (NLRC4), and NLRP3 (93). Among all the NLR molecules discovered, the NLRP3-inflammasome has been extensively studied in recent years. It can be activated by mitochondrial dysfunction, ionic influx, reactive oxygen species (ROS) production, and stimuli from pathogens or damaged cells (94). In general, sensitization of the NRLP3 inflammasome involves two steps. In the first step, dependent on NF-κB signaling, the production of NLRP3, pro-IL1β, and pro-IL18 is induced by other PRRs. In the second step, NLRP3 recruits the apoptosis-associated speck-like protein (ASC), which results in the formation of ASC prion-like oligomer that further binds to pro-caspase-1 and lead to its activation. Sequentially, activated pro-caspase-1 cleaves pro-IL1β and pro-IL18 into IL1β and IL18, respectively, to initiate the innate immune response and inflammatory reaction (8, 94).

NLRP3 plays a vital role in the inflammation caused by the virus. For example, it has been found that the levels of NLRP3 are low in normal liver tissues; however, activation of the NLRP3 inflammasome is implicated in hepatic injury. The NLRP3 inflammasome mediates liver failure by sensitizing procaspase-1 and pro-IL-1 β in HBV-related acute-on-chronic liver failure (ACLF) (95, 96). To date, the molecular mechanisms associated with virus-mediated sensitization of the NLRP3 inflammasome have not been well examined. However, a recent study by Ding et al. showed that the HBV core protein is capable of enhancing the lipopolysaccharide-induced activation of the NLRP3 inflammasome and promoting IL-1β production to cause liver inflammation (97). However, dependent on HBeAg, the virus is discovered to suppress LPS-induced activation of NLRP3 inflammasome and IL-1β production *via* inhibiting the sensitization of NF-κB pathway and the production of reactive oxygen species. The inhibition of NLRP3 inflammasome mediated by HBeAg may be responsible for HBV-mediated restrain of innate immune response (98).

Dependent on the activation of the NLRP3 inflammasome, HBX is found to promote hepatocyte pyroptosis by triggering the production of ASC, IL-1β, IL-18, and HMGB1 in hydrogen peroxide-stimulated hepatocytes (32) (**Figure 1**). Additionally, NLRP3 inflammasome sensitization stimulated by the viral protein may be related to liver inflammation caused by viral infection. Current evidence indicates that RIP1 contributes to the regulation of IL-1β maturation by activating the NLRP3 inflammasome. Xie et al. found that the overexpression of HBX increased the expression of RIP1 in hepatocytes. Mechanistically, AFB1 participates in the HBX-induced upregulation of RIP1 (99, 100). Although RIP1 contributes to the activation of AP-1 and NF-κB to induce inflammation in HBX-positive cells, its effect on NLRP3 inflammasome sensitization mediated by HBX has not yet been assessed; therefore, further investigation is required to assess the effect of RIP1 on the regulation of inflammasome activation. In addition to NLRP3, NLRC4 was also found to interact with HBX based on substrate-trapping proteomics analysis (101). However, whether HBX affects NLRC4-associated inflammasomes has not yet been well assessed.

Similar to NLRP3, the absent-in-melanoma-2 (AIM2) inflammasome can recognize cytoplasmic DNA, resulting in the production of IL-1β and IL-18 and the induction of target cell pyroptosis (102). Although the mRNA expression levels of AIM2 are upregulated in PBMCs of patients with acute and chronic hepatitis B (103), Chen et al. found that in HBV-related HCC cells, HBX not only suppressed the expression of AIM2 at the gene level by promoting the stability of the enhancer of zeste homolog 2 (EZH2), but also interacted with AIM2, resulting in AIM2 degradation *via* the ubiquitin-proteasome pathway. Functionally, knockdown of AIM2 enhances HBX-mediated migration and metastasis of hepatoma cells (104). Nevertheless, based on the current studies, whether HBX can influence the inflammasomes *via* AIM2 remains unclear. Moreover, the effect of HBX on the inhibition of AIM2-associated inflammasomes needs to be explored further in future studies.

## Role of HBX in the JAK-STAT Pathway

JAK-STAT signaling is a vital downstream pathway of IFN receptors that stimulates IFN-stimulated gene (ISG) production (8, 105). The JAK family comprises of JAK1-3 and TYK2. The STAT family has seven members, including STAT1-4, STAT5a, STAT5b, and STAT6 (106). JAK-STAT signaling is known to play a critical role in IFN-mediated inhibition of HBV replication (3). To facilitate the persistence of HBV infection, the virus can promote MMP-9 and CTHRC1 expression to repress JAK-STAT signaling (107, 108). HBeAg can also inhibit JAK-STAT signaling to enhance HBV replication (109).

The JAK-STAT pathway is a highly conserved signaling pathway that can affect various biological processes including immune response, apoptosis, inflammation, tissue repair, and adipogenesis (110). Currently, the link between HBX and sensitization of JAK-STAT signaling has been well established (**Table 1**). Current evidence shows that the regulation of JAK-STAT signaling activation mediated by HBX plays a vital role in not only regulating cellular proliferation, apoptosis, epithelial-mesenchymal transition (EMT), and migration, but also in modulating the innate immune response and viral replication. HBX regulates JAK1, JAK2, and TYK2. For example, HBX interacts with JAK1 to facilitate its activation (111). Furthermore, HBX-mediated JAK1 activation is associated with sensitization of the Ras-Raf1 signaling axis (112). In addition to JAK1, HBX also contributes to the activation of JAK2 to induce apoptosis of renal tubular epithelial cells (113). However, Cho et al. showed that HBX can inhibit the activation of TYK2 to decrease the expression of IFN-α receptor 1 (IFNAR1) to inhibit extracellular IFN-mediated signal transduction (114).

**Table 1:** The detailed information on the regulation of JAK-STAT signaling mediated by HBX.

Target molecules	The role of HBX on target molecules	The regulated molecules in JAK-STAT signaling	The role of HBX on JAK-STAT signaling	Biological processes	References
JAK1	interaction	JAK1	activation	The activation of Ras-Raf1 signaling axis	(111, 112)
JAK2	activation	JAK2	activation	Cell apoptosis	(113)
TYK2	inhibition	TYK2	inhibition	IFN-mediated signal transduction	(114)
STAT1	activation	STAT1	activation	Reoviral oncolysis of HCC cells	(115)
STAT3	activation	STAT3	activation	Apoptosis, complement-dependent cytotoxicity, mitochondrial association, EMT, Insulin signaling, tumorigenicity, self-renewal, drug resistance	(113, 116–123)
LINC0115 /IL23	upregulation	STAT3	activation	Cellular proliferation and survival	(124)
SH2D5	upregulation	STAT3	activation	Cellular proliferation	(125)
IL-6	upregulation	STAT3	activation	Liver regeneration, Tumorigenesis	(126, 127)
Lethal-7	inhibition	STAT3	activation	Cellular proliferation	(119)
LASP1	upregulation	STAT3	activation	EMT	(128)
STAT5b	activation	STAT5b	activation	EMT	(129)
IL-34	upregulation	STAT3	activation	Cellular proliferation and migration	(130)
HULC	upregulation	STAT3	activation	HBV replication, cellular proliferation	(131)

HBX also plays a vital role in STAT activation. Especially, among the STAT molecules, the effect of HBX on the sensitization of STAT3 has been wildly investigated. For example, viral proteins inhibit reoviral oncolysis of hepatoma cells by activating STAT1 (115). In human renal proximal tubular epithelial cells, HBX modulates apoptosis by activating the STAT3 signaling pathway (113). Additionally, HBX decreases nephrin expression and induces podocyte apoptosis by activating STAT3 (116). However, a recent study revealed that depending on STAT3, HBX protects hepatoma cells and hepatocytes from complement-dependent cytotoxicity by increasing the membrane-bound complement regulatory protein CD46 (117). In particular, HBX-mediated STAT3 activation may be associated with oxidative stress in HBV-associated hepatoma cells (118). In addition, HBX can increase the transcription of LncRNA LINC01152 to enhance IL-23 expression and then initiate the activation of STAT3 to promote the proliferation and survival of hepatoma cells (124). In HCC cells, HBX was also found to trigger SH2D5 expression, and based on the HBX-mediated interaction of SH2 domain-containing 5 (SH2D5) with transketolase (TKT), STAT3 can be activated to promote HCC cell proliferation (125). IL-6 plays a vital role in the activation of STAT3. Current research indicates that HBX can promote IL-6 expression in hepatoma cells (126). In response to IL-6, HBX recovers the dephosphorylation of STAT3 mediated by PP2Cα, a protein that interacts with HBX to facilitate hepatocarcinogenesis (127). Additionally, downregulation of miRNA Lethal-7 mediated by HBX also activates STAT3 to regulate cellular proliferation (119).

EMT has been implicated in HCC development. It has been demonstrated that HBX contributes to the activation of STAT3 to control the EMT of hepatoma cells (120). Depending on STAT3, HBX promotes the expression of HMGB1 to enhance EMT in liver cancer cells (121). Additionally, our results have shown that HBX can activate STAT3 through LASP1 to facilitate vimentin expression and enhance EMT (128). Similar to STAT3, STAT5b participates in the induction of EMT mediated by HBX (129).

Suppressor of cytokine signaling (SOCS) mediates insulin resistance in the liver. Kim et al. found that HBX could induce SOCS3 expression *via* STAT3 to impair hepatic insulin signaling (122). Up to now, more and more evidence has demonstrated that C-terminally truncated HBX contributes to the development of HCC (2). In particular, Ching et al. found that relying on STAT3, C-terminal truncated HBX can regulate tumorigenicity, self-renewal, and drug resistance (123). Additionally, our previous study showed that HBX was capable of promoting the levels of IL-34 to activate STAT3 and further facilitate the growth and migration of liver cancer cells (130).

Interestingly, although JAK-STAT signaling participates in the inhibition of HBV replication, the current study showed that STAT3 also contributes to HBX-mediated HBV replication. Long noncoding RNA (lncRNA) is highly upregulated in liver cancer (HULC) and has been identified to be significantly upregulated in HCC. Liu et al. found that HULC could elevate the expression of HBX, which in turn sensitizes STAT3 to stimulate the miR-539 promoter. miR-539 decreased the expression of APOBEC3B and then enhanced HBV replication. Furthermore, HULC mediated by HBX can also enhance the proliferation of hepatoma cells in *in vitro* and *in vivo* models (131).

## Influence of HBX on the NF-κB Pathway

As mentioned above, NF-κB contributes to the innate immune responses induced by different PRRs. In the cytoplasm, IκB-α and IκB-β bind to NF-κB p50/p65 and form an inactivated protein complex. Depending on the upstream signal transduction from the IKK complex, which is composed of IKKα, IKKβ, and IKKγ, IκB proteins are degraded to free NF-κB p65/p50. Next, NF-κB p65/p50 is transported into the nucleus, resulting in the transcription of different inflammatory cytokines (132, 133).

The published reports have suggested that the activation of NF-κB facilitates the inhibition of HBV replication (134–136). To accelerate replication, the virus promotes fibronectin expression to restrain the activation of NF-κB and sensitize HBV enhancers (137). Wang et al. showed that HBeAg can interrupt the ubiquitination of NEMO to suppress NF-κB activity and enhance HBV replication (138). HBeAg also suppresses NF-κB signaling mediated by IL-18 and IL-1β in natural killer (NK) cells and hepatocytes, and the inhibition of NF-κB signaling may contribute to the maintenance of persistent HBV infection (139, 140). However, current evidence from other groups has shown that NF-κB signaling also facilitates HBX-mediated HBV replication. For instance, HBX can promote HBV replication by inhibiting the miR-192-3p-XIAP axis to activate the NF-κB pathway (141). Xu et al. found that HBX can activate NF-κB to promote the expression of IFIT3 and then enhance viral replication (142). HBX increases gp96 expression *via* NF-κB signaling to facilitate HBV replication (143). HBX upregulates miR-146a-5p *via* NF-κB to increase autophagy and enhance HBV replication (144) (**Table 2**). To date, the reasons for the contradictory function of NF-κB signaling in viral replication in different studies are unknown and need to be explored in the future.

**Table 2:** The detailed information related to the modulation of NF-κB signaling mediated by HBX.

Target molecules	The role of HBX on target molecules	The regulated molecules in NF-κB signaling	The role of HBX on NF-κB signaling	Biological Functions	References
p65	activation	p65	activation	HBV replication	(142–144)
IkBα	inhibition	IkBα	activation	Signal transduction activation	(145)
p65	interaction	p65	activation	Inflammation, tumorigenesis	(146)
IKKγ	interaction	IKKγ	activation	Signal transduction activation	(86, 147)
IKKα	upregulation	IKKα	activation	Inflammatory cytokine production	(148)
IKKβ	activation	IKKβ	activation	Cellular proliferation	(149)
ECSIT	interaction	IKK α/β, IκBα, p65, p50	activation	Signal transduction activation	(75)
VBP1	interaction	Unknown	activation	Cellular proliferation	(150)
VCP	interaction	Unknown	activation	Signal transduction activation	(151)
AIB1	interaction	Unknown	activation	Signal transduction activation	(152)
PI3-K	activation	p50, p65, IKKα	activation	Cellular motility	(153, 154)
ERK	activation	p50, p65	activation	Inflammatory responses	(155)

In addition to viral replication, many studies have suggested that sensitization of NF-κB induced by HBX plays a vital role in cellular growth, apoptosis, EMT, and migration of hepatoma cells (156, 157). Mechanistically, HBX can interact with NF-κB signaling molecules to initiate the signal transduction activation (**Table 2**). For example, HBX can inhibit IkBα to induce sustained activation of this signaling pathway (145). In addition, HBX can bind to NF-κB (p65) to facilitate its activation and nuclear localization and induce the expression of metastasis-associated protein 1 (MTA1), which is involved in inflammation and tumorigenesis (146). Furthermore, the activation of NF-κB signaling induced by HBX is mediated by the interactions between HBX and IKKγ (86, 147). In addition, HBX can enhance the expression of IKKα and modulate the activity of IKKβ to activate NF-κB signaling to facilitate inflammatory cytokine production and cell proliferation (148, 149).

In addition, accumulating evidence has also verified that multiple molecules participate in NF-κB activation initiated by HBX (**Table 2**). HBX enhances NF-κB activation by interacting with ECSIT (75). HBX can directly interact with VHL-binding protein (VBP1) and synergistically promote NF-κB activation to facilitate cellular proliferation (150). Viral proteins can enhance the activation of NF-κB through interactions with the valosin-containing protein (VCP) (151). In addition, HBX binds to amplification in breast cancer 1 (AIB1) protein and stabilizes the protein to enhance the sensitization of NF-κB signaling (152). Additionally, the reports from Lim et al. showed that dependent on the chaperoning activity, ribosomal protein S3α (RPS3α) can stabilize HBX and enhance the activation of NF-κB signaling (158).

In recent years, increasing evidence has shown that HBX can influence various signaling pathways to initiate hepatocarcinogenesis. Interestingly, HBX can activate NF-κB through a variety of distinct signaling pathways involving PI3-K (153, 154), and ERK (155), contributing to the regulation of motility and inflammatory responses in HCC cells. Autophagy is a vital metabolic process that is dependent on the degradation of damaged proteins and organelles in lysosomes and can maintain homeostasis of the intracellular environment. Luo et al. suggested that HBX can induce autophagy to stimulate the activation of NF-κB in hepatocytes (148).

## Conclusions

Although the innate immune system plays a central role in targeting HBV, the virus triggers only a small innate response and has evolved a variety of escape strategies to block the antiviral response in host cells. As a viral nonstructural protein, HBX is considered a vital therapeutic target for HBV infection because the viral protein not only contributes to the replication of the virus and participates in the development of liver cancer induced by HBV, but also protects HBV-infected cells from immune-mediated clearance (22, 159). In the present review, we summarized the recent progress regarding the role and associated mechanisms of HBX in PPR signaling during HBV infection. Our reviewed studies indicate that HBX can regulate the expression and function of various vital molecules in TLR, RLR, and NLR signaling, as well as their downstream pathways, including the JAK-STAT and NF-κB pathways. Interestingly, the effect of molecules in these signaling pathways mediated by HBX is different during HBV infection. First, many molecules involved in TLR signaling can be regulated by HBX to inhibit the immune response, aid in viral replication, and regulate a variety of biological processes to modulate inflammation and facilitate the development of HCC. Second, regulation of RLR signaling mediated by HBX can inhibit the immune response to benefit viral replication. Third, HBX may promote the activation of NLR signaling to cause inflammation during HBV infection. Fourth, the regulation of the JAK-STAT and NF-κB pathways stimulated by viral proteins controls viral replication and modulates different biological processes to accelerate the development of HCC. In particular, current data show that targeting HBX could suppress viral replication by enhancing the immune response mediated by PRR signaling in HBV-expressing liver cells (160–162). Additionally, inhibition of innate immune-related molecules regulated by HBX also blocks HCC development (29). Therefore, a deeper understanding of the regulation of PRR signaling by HBX may facilitate the treatment of HBV infections and related illnesses.

Cyclic GMP-AMP synthase (cGAS) and DNA-dependent activator of IFN regulatory factors (DAI) participate in DNA-dependent immune responses (163, 164). In particular, after cytoplasmic DNA is activated by cGAS or DAI, these molecules can induce the activation of the stimulator of interferon genes (STING) to further induce the expression of IFN and inflammatory cytokines (8, 165). It has been found that the cGAS-STING pathway facilitates the inhibition of HBV replication and attenuates hepatocyte injury and fibrosis induced by the virus (166–168). However, the expression levels of cGAS and its effector genes have been shown to decline in hepatocytes infected with HBV (169). Additionally, STING expression levels in peripheral monocytes were dramatically decreased in patients with CHB (170). To maintain persistent infection, the virus can evade the antiviral activity of the cGAS-STING pathway by various strategies (166, 171). For example, HBV polymerase protein can decrease the ubiquitination of STING and inhibit the production of IFN by interacting with STING (172). HBsAg can inhibit STING expression and suppress the immune response of NK cells (173). HBX has also been found to restrain the dsDNA-induced immune response (30) and suppress STING-mediated activation of IFN-β (31). However, the cellular factors that contribute to the suppression of STING-associated signaling mediated by HBX have not yet been well elucidated. Given the importance of STING-mediated immune response in the restriction of HBV (171), further studies are needed to assess the effect of regulation of HBX on the immune response mediated by STING in the future.

## Author Contributions

All authors listed have made a substantial, direct, and intellectual contribution to the work, and approved it for publication.

## Funding

The study was supported by Xuzhou Technology Bureau Foundation (KC21065), the Natural Science Foundation of Jiangsu Province (BK20211347), the Natural Science Foundation of the Jiangsu Higher Education Institutions (21KJA310004), Suqain Sci&Tech Program (K202015), and a project funded by the Priority Academic Program Development of Jiangsu Higher Education Institutions (PAPD).

## Conflict of Interest

The authors declare that the research was conducted in the absence of any commercial or financial relationships that could be construed as a potential conflict of interest.

## Publisher’s Note

All claims expressed in this article are solely those of the authors and do not necessarily represent those of their affiliated organizations, or those of the publisher, the editors and the reviewers. Any product that may be evaluated in this article, or claim that may be made by its manufacturer, is not guaranteed or endorsed by the publisher.
